# The efficacy of etodolac and ibuprofen, regarding gender, on pain, edema and trismus after impacted lower third molar surgery: A randomized prospective clinical split-mouth study

**DOI:** 10.4317/medoral.24082

**Published:** 2020-10-09

**Authors:** Leonardo de Freitas Silva, Erik Neiva Ribeiro de Carvalho Reis, Leonardo Perez Faverani, Ana Paula Farnezi Farnezi Bassi

**Affiliations:** 1 DDS, MSc and PhD in Oral and Maxillofacial Surgery. Department of Surgery and Integrated Clinic, Araçatuba Dental School, UNESP, Araçatuba, São Paulo, Brazil; 2 DDS, MSc in Oral and Maxillofacial Surgery. Department of Surgery and Integrated Clinic, Araçatuba Dental School, UNESP, Araçatuba, São Paulo, Brazil

## Abstract

**Background:**

This study aimed to conduct a randomized prospective study about the efficacy of etodolac and ibuprofen on trismus, pain and edema regarding gender of patients submitted to impacted lower third molar teeth extraction.

**Material and Methods:**

Thirty patients aging between 16 and 35 year-old were submitted to the exodontia of impacted lower third molars. During the postoperative period, patients received nine ibuprofen (600 mg) or etodolac (300 mg) pills via oral administration immediately after surgery and repeated doses every eight hours during three days. Patients were evaluated regarding pain, trismus and edema.

**Results:**

Sixteen men and fourteen women participated of the study. No statistical difference was established regarding gender according to the evaluated parameters. However, etodolac use showed better results regarding pain, trismus and edema.

**Conclusions:**

Pain, edema and trismus after impacted third molars extraction were not influenced by gender.

** Key words:**Molar, third; sex, tooth extraction.

## Introduction

Impacted third molar teeth removal is the most executed oral surgery procedure (1-3). However, this procedure is associated to postoperative complications, such as pain, edema and trismus (1-4). The control of these symptoms is frequently based on pharmacological manipulation of local and systemic pain and inflammation mediators (3).

Postoperative pain is related to alterations in the central and peripheral nervous systems induced by trauma (5). This injury causes liberation of cyclooxygenase-2 (COX-2), which induces prostaglandins activity, promoting peripheral nociceptors sensitization and inflammatory symptoms emergence (6).

Clinical and experimental researches indicate that pain is perceived, evaluated and treated differently depending on gender, race/ethnicity and age of a person (7). When compared to men, women report more pain and present lower threshold and tolerance to experimental pain stimuli (8-10).

Although, there are studies in the literature about pain perception regarding gender, there are still no studies about pain comparing genders after impacted third molars removal. Thus, this study aimed to execute a randomized prospective study about the efficacy of etodolac and ibuprofen on trismus, pain and edema according to the gender of patients submitted to impacted lower third molars extraction.

## Material and Methods

This study was approved by the Research Ethics Committee of Araçatuba Dental School – FOA UNESP, under protocol number 818.680. Every patient signed the authorization term to the execution of the study and disclosure of results according to the medical and ethical protocol of the Declaration of Helsinki, 2013.

Thirty healthy patients aging between 16 and 35 year-old participated of this study. Patients selection followed the adopted inclusion criteria: absence of systemic health problems and good oral hygiene; presence of bilateral impacted lower third molar teeth with extraction indication through ostectomy and odontostomy; teeth of both sides should present the same surgical difficulty level evaluated by panoramic radiographs, and present at least two thirds of the roots formed.

Exclusion criteria for this study included smokers; systemic disease patients, such as diabetes, osteoporosis, hyperthyroidism, pregnant and lactating women; patients with hypersensitivity to ibuprofen, etodolac or dexamethasone; patients presenting third molars with different surgical difficulty levels between right and left sides; periodontitis; local infection or cysts and tumors.

Sample size was obtained through the power of a test accomplished at the website www.lee.dante.br/pesquisa/amostragem/amostra, which indicated that a sample with nine patients per group would reach a power of 0.8. Thus, thirty patients were selected to participate of the study. Patients had both lower third molar teeth removed by the same surgeon with time interval of 21 days between both surgical procedures.

Each patient took 4 mg dexamethasone one hour before the procedure. After the removal of the right or left lower third molar tooth, randomly chosen by the research assistant, each patient received 600mg of ibuprofen (Brainfarm, Anápolis, Brazil) or 300mg of etodolac (300 mg; Flancox; Apsen, Santo Amaro, Brazil) orally every 8 hours for 3 days, starting immediately after surgery. Each third molar tooth was removed in different moments with time interval of at least 21 days. Patients who received ibuprofen after the first surgery, received etodolac after the second surgery; and those who received etodolac after the first surgery, received ibuprofen after the second. All patients received 500 mg paracetamol pills to take every six hours in case of pain.

The choice between right or left sides in the first procedure was defined using the website www.randomization.com, and both, surgeon and patient, did not know which medication was administered to the chosen side. The assistant was responsible for collection of data regarding the operated side and administered medication.

- Surgical procedure

After intraoral antisepsis using aqueous solution of 0.12% chlorhexidine gluconate and extraoral antisepsis with alcoholic solution of 0.5% chlorhexidine, the surgeon executed regional block of the inferior alveolar, lingual and buccal nerves using 4% articaine with 1:100,000 epinephrine (DFL®, Rio de Janeiro, Brazil).

After local anesthesia, the surgeon made a full-thickness mucoperiosteal flap extended posteriorly to the second molar, and a relaxing incision in the vestibular region anteriorly to the same tooth. Flap detachment was executed using a Molt Periosteal Elevator No. 9 (Quinelato®, São Paulo, Brazil), ostectomy and odontostomy were made using an Oral Surgery Bur 702 (KG Sorensen®, São Paulo, Brazil) connected to a high speed handpiece (KaVo Dental Brazil Ltda, Santa Catarina, Brazil) under constant irrigation with saline solution.

Teeth were removed using dental extraction forceps and the surgical wound closure was made using silk suture 4.0 (Ethicon®, Johnson e Johnson, São Paulo, Brazil) with interrupted stiches. Suture was removed after 7 days.

Postoperative care included eating cold soft foods and liquids within the first 48 hours, rest and mouthwash only after 24 hours. Patients who presented complications, such as bleeding, alveolitis or infection were excluded from the study. Patients were reassessed from 48 to 168 hours (seven days) by a third examiner who was not aware which group the patient belonged to. Pain, edema and trismus were evaluated.

- Variable analysis

Pain: Pain level was evaluated through BS-11 analogue scale for pain (11). This scale uses a line, in which tips represent the limits of pain, varying from absent to the worst imaginable pain. Patients were oriented to write down the pain level into pre-determined blank spaces within the line, using numbers from 0 (absent) to 10 (worst pain). After each surgical procedure, patients received an analogue scale to write down pain severity 6, 12 and 24 hours after surgery, as well as the number of painkillers taken.

Edema: Before each surgical procedure, patients’ face profiles were measured through the distance between the tip of the chin (reference: area between the lower central incisors) and the lower portion of the earlobe, using a measuring tape (12). Data was recorded before surgery, and 48 and 168 hours (7 days) after surgery.

Measurements were taken with patients in maximum intercuspation and relaxed lips. Each measurement was repeated 3 times in order to increase reliability of the method (13). Spots in the lower region of the earlobe and chin were marked with a skin tattoo pen (Henafix, Belo Horizonte, Brazil), and these marks remained on the skin for 10 days.

Trismus: Trismus was evaluated by measuring maximum mouth opening using a digital pachometer (Mitutoyo, Japan), references used were the incisal edges of teeth 11 and 41. Measurements were taken before surgery, and 48 and 168 hours (7 days) after surgery.

- Statistical Analysis

Statistical analysis was conducted using SigmaPlot software (version 13.0; Systat Software, San Jose, CA). Shapiro-Wilk test was used to evaluate the normality of data distribution, followed by parametric and nonparametric tests application. Three-way analysis of variance was used to measure pain, trismus, edema and gender. Statistical significance considered was *p*<0,05.

## Results

Thirty healthy patients, 16 men and 14 women, aging between 16 and 35 year-old participated of this study. Patients attended postoperative evaluations and no complications occurred during this period.

**Figure 1:**
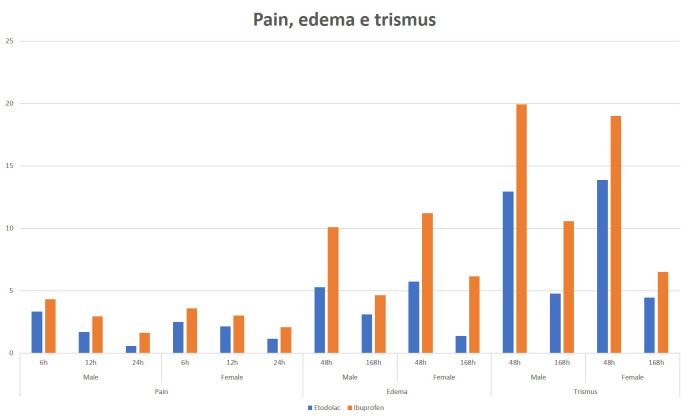
Pain, trismus and edema according to gender and medication used.

Regarding pain, etodolac administration provided better comfort to patients, showing lower pain values than ibuprofen in all postoperative periods (Fig. 1). This difference was statistically significant (Table 1). Regarding gender, no statistical difference was observed within groups (Fig. 1 and Table 1).

In both, ibuprofen and etodolac groups, edema peak happened in the first 48 hours, as expected. The group using etodolac presented lower edema coefficient than ibuprofen group (Fig. 1). No statistical difference was observed regarding gender (Fig. 1 and Table 2).

Regarding trismus, the lowest values occurred in the group using etodolac (Fig. 1), the difference was statistically significant (Table 3). Regarding gender, no statistical difference was observed (Fig. 1 and Table 3).

**Table 1:**
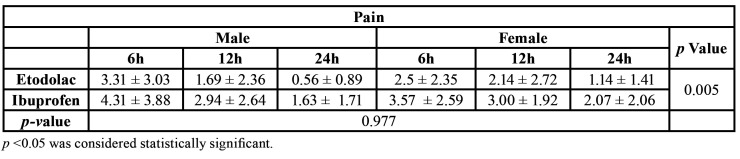
Mean of values obtained for pain.

**Table 2:**
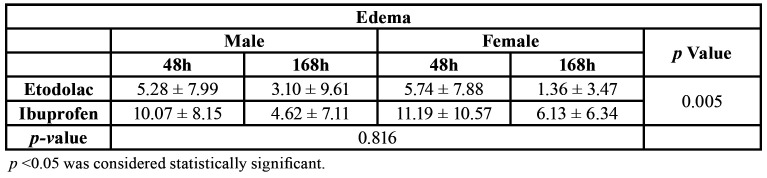
Mean values obtained for the edema coefficient.

**Table 3:**
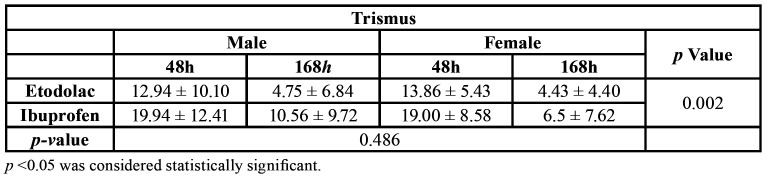
Mean values obtained from the trismus coefficient.

## Discussion

Postoperative pain after impacted third molar teeth surgeries has become a model used by studies on acute pain (14), because these surgical procedures can be easily classified and data about pain after teeth extraction can substantiate clinical tests sensitivity, thus, it is useful to predict the analgesic efficacy of non-steroidal anti-inflammatory drugs (15,16).

According to a review by Fillingim *et al*. (9) about painful sensation regarding gender, they observed that there is a lack of standardization in the measures used or in the period of time to evaluate postoperative pain, besides, there are no studies on population level for this sort of pain. In the literature, there are studies evaluating post-surgical pain in cholecystectomy (17), colonoscopy (18), total hip arthroplasty (19), knee arthroplasty (20), arthroscopic knee repair (21), and outpatient surgeries (22,23). Thus, the lack of studies evaluating pain difference among genders in oral surgical procedures motivated this study.

A study by Bjørnnes *et al*. (24) regarding pain difference between genders concluded that women reported more pain than men up to one year after cardiac surgery. Authors suggest that data indicate that women were more afraid to use analgesic drugs than men and they were more concerned to report pain (24).

Chia *et al*. (22) studied 2,298 patients submitted to outpatient surgeries and observed that men presented more painful sensation than women during rest or movement. Besides, women used less morphine than men (22).

In this study, female gender presented less pain sensitivity than men during postoperative period within the first 6 hours, different from the period of 12 and 24 hours after surgery. However, there was no statistical difference between groups.

Regarding medication used, Bailey *et al*. (25) conducted a literature review comparing ibuprofen and paracetamol use for pain relief after third molar teeth removal. Authors observed that ibuprofen is superior to paracetamol on doses of 200-512mg and 600-1,000mg (25). Best *et al*. (26) observed that the addition of codeine to the association of ibuprofen with paracetamol did not present better results on pain after third molar teeth extraction (26). In this study, opioid analgesics were not used to postoperative pain relief. Besides, paracetamol was prescribed for patient use only in case of pain, in accordance to the literature.

Akbulut *et al*. (1) compared the use of diclofenac, naproxen and etodolac to pain control, edema and trismus after third molars extraction surgery (1). Authors did not observe difference regarding pain and trismus, although, diclofenac presented better results on edema reduction (1).

In this study, etodolac showed better results regarding edema, pain and trismus in all periods evaluated. No statistical difference was observed regarding edema and trismus between genders, presenting similar results between groups.

This is a randomized mouth-split clinical trial with a reliable methodology in order to evaluate pain perception difference among genders. It was possible due to the standardization of third molar teeth removals, quantification of pain through a method published in the literature and, besides that, this is an unprecedented study due to the lack of studies published in the literature. However, this study presents some limitations regarding pain evaluation. Pain is subjective and is related to gender, age and ethnicity. Besides, other factors may influence pain perception, such as individual threshold and tolerance, and emotional state of the person. However, these limitations do not invalidate the study, because it is not possible to achieve this total control level in clinical trials.

This study concluded that pain, edema and trismus after impacted third molar teeth extraction were not influenced by gender. Regarding the medication used, etodolac presented better postoperative results for the evaluated parameters. Despite results found, more studies are necessary to determine the role of gender in the postoperative period of oral surgery.
